# Boosting tissue-specific prediction of active cis-regulatory regions through deep learning and Bayesian optimization techniques

**DOI:** 10.1186/s12859-022-04582-5

**Published:** 2022-12-12

**Authors:** Luca Cappelletti, Alessandro Petrini, Jessica Gliozzo, Elena Casiraghi, Max Schubach, Martin Kircher, Giorgio Valentini

**Affiliations:** 1 AnacletoLab, Dipartimento di Informatica, Università degli Studi di Milano, Milan, Italy; 2 Berlin Institute of Health at Charité, Universitätsmedizin Berlin, Berlin, Germany; 3 European Laboratory for Learning and Intelligent Systems (ELLIS), Berlin, Germany; 4 CINI National Laboratory of Artificial Intelligence and Intelligent Systems (AIIS), Rome, Italy; 5 Data Science Research Center, Università degli Studi di Milano, Milan, Italy

**Keywords:** Neural networks, Deep learning, Prediction of cis-regulatory region, Bayesian optimization

## Abstract

**Background:**

Cis-regulatory regions (CRRs) are non-coding regions of the DNA that fine control the spatio-temporal pattern of transcription; they are involved in a wide range of pivotal processes such as the development of specific cell-lines/tissues and the dynamic cell response to physiological stimuli. Recent studies showed that genetic variants occurring in CRRs are strongly correlated with pathogenicity or deleteriousness. Considering the central role of CRRs in the regulation of physiological and pathological conditions, the correct identification of CRRs and of their tissue-specific activity status through Machine Learning methods plays a major role in dissecting the impact of genetic variants on human diseases. Unfortunately, the problem is still open, though some promising results have been already reported by (deep) machine-learning based methods that predict active promoters and enhancers in specific tissues or cell lines by encoding epigenetic or spectral features directly extracted from DNA sequences.

**Results:**

We present the experiments we performed to compare two Deep Neural Networks, a Feed-Forward Neural Network model working on epigenomic features, and a Convolutional Neural Network model working only on genomic sequence, targeted to the identification of enhancer- and promoter-activity in specific cell lines. While performing experiments to understand how the experimental setup influences the prediction performance of the methods, we particularly focused on (1) automatic model selection performed by Bayesian optimization and (2) exploring different data rebalancing setups for reducing negative unbalancing effects.

**Conclusions:**

Results show that (1) automatic model selection by Bayesian optimization improves the quality of the learner; (2) data rebalancing considerably impacts the prediction performance of the models; test set rebalancing may provide over-optimistic results, and should therefore be cautiously applied; (3) despite working on sequence data, convolutional models obtain performance close to those of feed forward models working on epigenomic information, which suggests that also sequence data carries informative content for CRR-activity prediction. We therefore suggest combining both models/data types in future works.

## Background

Non-coding DNA regions, which account for 98% of the whole human genome, were regarded as “junk DNA” in the past. However, their importance is now established in the scientific community, given the discovery of non-coding cis-regulatory regions (CRRs) that regulate the transcription of neighbouring genes, therefore determining spatio-temporal patterns of gene expression [1, 2].

Indeed CRRs are involved in the development of different tissues and/or cell types, in the definition of gene expression patterns during the cell life, e.g. by determining the precise moment of transcription and its intensity, and in the dynamical response to changes in physiological conditions [3].

Genome-wide association studies (GWAS) discovered thousands of variants associated with diseases and traits enriched in non-coding sequences [4, 5]. These results have been confirmed and refined by recently proposed machine learning methods for the detection of deleterious and pathogenic variants in non coding regions [6–8], as well as by other research works that showed that cis-regulatory variants are involved in both common and rare human diseases [9–11].

Based on the aforementioned studies, an essential research question for understanding the functional impact of genetic variants on human diseases, as well as the mechanisms underlying the modulation of gene expression, regards not only the identification of CRRs, but also their activation status, which is specific to each cell type [12, 13] and is one of the key mechanisms for cell type differentiation [14]. In other words, genetic variants in tissue-specific CRRs show a different effect depending on the activity of the CRRs where they are located. Indeed, variants occurring in active CRRs can exert their full potential deleterious or pathogenic effect when they are located in tissue-specific active regulatory regions.

To advance knowledge about the identification of cis-regulatory elements in different cell types or tissues [15, 16], and to map TF binding sites and histone modifications across cell types and tissues, several lines of research have been proposed, which exploit multiple high-throughput technologies [17–21]. These experiments resulted in notable projects (the ENCODE project [19], the FANTOM project [22], and the Roadmap Epigenomics Project [23], see “Related work” section) specifically aimed at identifying CRRs in different tissues and cells lines, mapping their epigenomic landscape.

However, the experimental identification of CRRs requires approaches that are expensive and time-consuming, and researchers are still far from obtaining a comprehensive map of CRRs across all cell types, disease statuses and developmental stages. This problem paved the way to a novel research line, where machine learning (ML) techniques are specifically developed to identify the location of enhancers and promoters and their activity status (active versus inactive). ML techniques represent a crucial tool for this task, given the successful results obtained by ML models in problems where human reasoning has difficulties in reaching a promising solution [24]. In a first attempt to tackle these tasks, initial approaches applied unsupervised learning techniques [25, 26], driven by the limited availability of reliable annotations that were insufficient to guide a supervised learning approach. Unfortunately, the simplicity of the exploited techniques did not allow to achieve acceptable enhancer prediction results (accuracy around $26\%$) [27], so that the interest switched towards boosted supervised learning models [28], and random-forest classifiers [29]. Subsequently, when the FANTOM5 Consortium [30] published large-scale and high-resolution CRRs locations [31], ensembles of support vector machines were proposed [32], and opened the way to the usage of more advanced models such as deep neural networks (see e.g. [33, 34]) which are able to uncover the underlying information and high-level patterns hidden by complex multi-dimensional manifolds.

In this study, we extend our preceding work [35], and build upon two relevant state-of-the-art works (see “Related work” section), named DECRES [34] and DeepEnhancer [33], respectively. In DECRES [34] authors applied deep learning models [36] to CRR-activity prediction tasks, and reduced negative effects due to high data unbalancing by a peculiar data re-sampling strategy, where also the test set is re-balanced (“Effect of different dataset-balancing setups” section).

DeepEnhancer [33] leverages Convolutional Neural Networks (CNN, “Methods” section), and obtained promising performance by processing one-hot-encoded sequence data for recognizing enhancers against background sequences, which highlighted the feasibility of sequence-based deep learning classifiers.

Our experiments on CRRs activity prediction are primarily aimed at: (1) investigating the influence of the model selection phase on the obtained performance; (2) understanding whether genomic sequences allow to obtain reasonable CRR activity prediction results; (3) understanding the effects of different data re-balancing strategies.

Indeed, as clarified at the beginning of “Methods” section, the design, development and training of deep neural networks is a challenging task driven by a high number of (architectural, as well as training) hyperparameters [36, 37] whose setting influence not only the models’ computational complexity but also their performance [37]. Therefore, the model selection phase is a crucial step in the design of deep neural models, and several automatized optimization algorithms have been proposed (“Related work” section), among which “Bayesian Optimization” proved its efficiency and effectiveness [38–40].

In particular, we applied Bayesian optimization to find the optimal architecture and hyperparameters of two deep neural network meta-models (a Feed-Forward Neural Network—FFNN-model, and a CNN model) and we comparatively evaluated: (1) the optimized models versus their fixed counterparts; (2) the FFNN models versus the CNN models; (3) the CNN models and their most related literature model (DeepEnhancer [33]); (4) the results obtained when performing/avoiding train and/or test set re-balancing.

Our results show that: (1) model selection by Bayesian optimization has the potential of improving performance, when using both genome version hg19 (GRCh37) and genome version hg38 (GRCh38); (2) sequence data analyzed through CNN models achieve results close to those obtained by FFNN trained on epigenomic data, therefore suggesting that also sequence data carries fundamentally informative content; (3) training and test set balancing should be cautiously performed since they can introduce biased or overoptimistic results.

The paper is organized as follows: in “Related work” section we report state-of-the-art methods strongly related to our approach; in “Results” section we report results computed by the deep learning models described in the “Methods” section when applied to the datasets detailed in the “Datasets” section, and by using the experimental setup described in the “Experimental setup” section. The “Discussion” section summarizes and critically analyzes the main results of our research. Concluding remarks and future perspectives are reported in the “Conclusions” section.

### Related work

In the last years, several projects highlighted that cell differentiation in humans is heavily controlled by the complex interaction of promoters and enhancers (jointly referred to as CRRs), which generally act by the binding of regulatory proteins (transcription factors).

Given their key role in human diseases [41], CRRs have been object of thorough investigation by genomic studies and biochemical experiments using high-throughput technologies. More precisely, experimentation in this field involves the detection of epigenomic features (e.g. TFs binding, presence of histone modifications, open chromatin regions, etc) which are associated with functional non-coding regions for inferring candidate cis-regulatory elements. Thanks to a fast and consistent decrease of the cost of these analytic methods, several consortia, including ENCODE [19], collected and aggregated the results of biochemical assays that used a wide range of high-throughput technologies. This resulted in large publicly available databases which now contain over one million putative enhancers over 147 cell types. These resources raised the research interest devoted to the development of new in-silico methods for the identification of CRRs, as well as their tissue-specific activity level and the possible impact of variants occurring in them. In particular, the FANTOM Project used CAGE (Cap Analysis of Gene Expression) technologies to map transcription initiation sites in 1816 human and 1016 mouse samples [22, 31]. The ENCODE and FANTOM projects differ for the kind of data they provide. ENCODE leveraged a massive array of genomic assays to capture transcriptomic and epigenomic data. Conversely, FANTOM focused mainly on the transcriptome by exploiting CAGE assays, relying on other published works to infer features like chromatin status [42]. Another important project, the Roadmap Epigenomics [23] considered 111 representative primary human tissues and cells and provided their epigenomic description, similarly to the ENCODE effort. A list of currently available databases for learning and understanding gene expression regulation is available in [15].

Recently, several deep learning models achieved state-of-the-art prediction results in different studies regarding regulatory regions in the human genome [33, 34, 43]. In particular, DeepEnhancer [33] (see “DeepEnhancer” section) uses CNNs to identify cell-specific enhancers from only sequence data; BiRen [44] similarly uses a hybrid deep learning architecture that integrates a gated recurrent unit-based bidirectional recurrent neural network and a CNN to predict human and mouse enhancers from sequence data.

Considering that approaches employing only DNA sequence do not take into account the regulatory mechanisms encoded in the epigenomic data (e.g. the state of the chromatin structure), PEDLA [43] predicts enhancers by using an extensive set of heterogeneous data, comprising different epigenomic, sequence, and conservation data, and a novel hybrid architecture integrating a deep neural network and a hidden Markov model. Interestingly, PEDLA iteratively learns from 22 training cell types/tissues and achieves high accuracy when predicting across 20 independent test cell types/tissues, showing high and consistent generalization performances across samples.

To improve the aforementioned methods by explicitly taking into account whether CRRs are active in the considered cell-lines/tissues, DECRES [34] labels the activity of CRRs by using annotation data extracted from FANTOM [30], and uses a wide set of epigenomic features from ENCODE [19], CpG islands, and phastCons evolutionary conservation scores [45] as input of several deep learning models, among which FFNN models, to identify not only the presence of enhancers and promoters, but also if they are active in a specific human cell-line. DECRES not only outperformed state-of-the-art unsupervised methods in all the considered tasks, but also allowed extending the FANTOM enhancer atlas by adding 16,988 bidirectionally transcribed loci, which allowed creating the so far most complete collection of CRRs in the human genome. Though interesting, DECRES exploits an experimental set-up where both the training and the test sets are balanced, which is quite uncommon. Indeed, when treating highly unbalanced sets, the training set is generally balanced to avoid the creation of a model which overfits the over-represented class, but the test set is kept unbalanced to avoid biasing the performance estimation.

When developing a classifier model and, in particular, a neural network, a crucial step regards the model selection task, that is, the choice of the specific neural network architecture (the number of hidden layers, their respective number of neurons, and the activation functions for each layer) and the setting of the learning hyperparameters (e.g. the optimizer algorithm, batch size, learning rate, and so on).

Though different automatic model selection techniques have been presented in literature (e.g. greedy search [46], sequential search [47], random search [48], grid search [49], particle swarm optimization approaches [50], genetic programming approaches [51], “Spectral approach” [52]), no well-accepted and unified method has been defined. For this reason, model selection is generally performed manually, by relying on past experiences, or empirically by consecutive tests, or automatically, by applying one of the aforementioned approaches to explore the hyperparameter space in a bounded domain, i.e. to search for the setting that minimizes (or maximizes) a user-defined objective function estimating the learner performance. One such approach is “grid search” which exhaustively evaluates the objective function for every possible hyperparameter combination in the bounded domain of the search space. Although being effective and highly parallelizable, grid search suffers from major drawbacks such as high computational costs exponentially increasing with the dimensionality of the hyperparameter space. For this reason, alternative approaches have been proposed in recent years. For instance, “random search” [48] efficiently explores the hyperparameter space by evaluating a sequence of randomly extracted points. The “spectral approach” [52] applies the Fourier transform to search the maximum or minimum of the objective function in the frequency domain.

Another well-known approach is Bayesian optimization [38–40], which efficiently exploits Bayes theorem to direct the search towards a (local) minimum/maximum of an objective function (the a posteriori estimation) that is often expensive to be optimized. Briefly, Bayesian optimization assumes a prior distribution of the loss function, and this prior is constantly updated by evaluating new observations. New points are selected by a proper pivot function called “Acquisition function” which regulates the criteria of “exploration versus exploitation”, so that the evaluation of the new point will provide a better overlook of the loss function (exploration) or a better identification of a maximum/minimum (exploitation). More details about Bayesian optimization are reported in “Bayesian optimization” section.

Due to the promising results achieved by applying Bayesian optimization to complex black box optimizations [53–55], and given its lower computational time when compared to grid search or random search, we have used it for the automatic selection of our classification models, which are described in the following sections.

## Results

In this Section we firstly overview the experiments using the FFNN models (Section *fixed-FFNN* and *Bayesian-FFNN*) and the CNN models (Section *fixed-CNN* and *Bayesian-CNN*) processing the dataset for genome version hg19/GRCh37 (hg19 dataset, detailed in the “Hg19-dataset” section, Table 1-top), which allowed to: (1) show that model selection through Bayesian optimization improves performance (“Bayesian optimization improves prediction of active regulatory regions" section) and that CNN models trained on sequence data obtain promising performance; (2) perform a comparison between our Bayesian CNN model trained on DNA sequence data and with the DeepEnhancer [33] state-of-the art model (“Bayesian CNN is competitive with DeepEnhance” section); (3) show the effect of different balancing setups (“Effect of different dataset-balancing setups” section). Next, in “Bayesian optimization also improves performance on hg38-dataset" section we report the FFNNs and CNNs performance obtained by experiments on the dataset for genome version hg38/GRCh38 (hg38-dataset, detailed in “Hg38-dataset” section, Table 1-bottom), which allows validating the model selection effectiveness on a wider sample set available for this genome assembly.

In the remaining part of this work, the regions considered in the following experiments, and detailed in “Datasets” section will be denoted as IE and AE for inactive and active enhancers, IP and AP for inactive and active promoters, and ELSE for regions which are either active (AX) or inactive exons (IX) or unknown/uncharacterized regions (UK), respectively.

### Experimental setup

In our experiments, we used both the hg19 and hg38 human genome reference assembly. We firstly run our experiments on the hg19 datasets provided by the authors of [34] (“Hg19-dataset” section) to allow a fair comparison with respect to their state-of-the-art work. Next, considering that several works in the biomedical field have transitioned to the hg38 assembly, and wishing to provide a more robust and reliable evaluation of the methods we are proposing, we also analyzed the hg38 datasets (“Hg38-dataset” section).

Following the above-mentioned notation, the three experiments on the hg19 dataset replicate the five binary classification tasks proposed in [34]: (1) IE versus IP, (2) AP versus IP, (3) AE versus IE, (4) AE versus AP, (5) AE + AP versus ELSE, by using FFNN models processing the epigenomic features provided by the authors [34], and CNN models processing one-hot-encoded sequence data. We then replicated the same experiments with the hg38-dataset, using sequence, epigenetic and labelling data for the hg38 human genome assembly. In this last setting, the fifth prediction task (AE + AP versus ELSE) was modified, so as to use only CRRs (AE + AP versus IE + IP) and avoid using regions very different from CRRs (AX, IX and UK), to potentially ease the recognition task.

Table 2 shows the unbalancing ratios for each prediction task on hg19 datasets (top), with average over the four classes ranging from 2.57 (IE versus IP) to 22.32 (AE versus IE), which differ from those for the hg38-dataset (bottom), with an average unbalance over the three classes from 1.57 (IE versus IP) to 7.58 (AE versus IP).

All the models were trained and tested by using random stratified holdouts with an $80/20$ split, that is, $80\%$ of each class was used as the training set and the remaining $20\%$ was reserved for the test set. We have executed $10$ holdouts for the experiments on the hg19 dataset and extended the holdouts number to $20$ for the experiments on the hg38 dataset. Where not otherwise stated, the prediction tasks were executed without executing data-balancing steps, where neither the training set nor the test set were re-balanced, so that their original distribution is maintained. The data-balancing is used exclusively when reproducing the experimental setup from [34].

Model selection through Bayesian optimization was performed on the training set by using additional stratified internal holdouts, with a train/validation ratio of $80/20$. With this setting, Bayesian Optimization aims at maximizing performance (measured by AUPRC) on the validation sets.

Before processing, the epigenomic data are normalized using “MinMax” scaling between 0 and 1.

We measured performance by using the Area Under the Receiver-Operating Curve (AUROC) [56] and the Area Under the Precision-Recall Curve (AUPRC) [57] over all the test sets in the holdouts. While AUROC is a standard performance evaluation metrics in machine learning, AUPRC was added because this performance metric is more appropriate when dealing with unbalanced datasets [58–60].

The statistical validation of the performance comparison (AUROC or AUPRC) of two different models, when applied to the same train/test holdouts, was performed by using the one-sided Wilcoxon signed rank-test at a 0.01 significance level (i.e. 99% confidence level or *p* value $p<0.01$) [61–63].

**Table 1:** For each dataset (on columns) we report the number of samples per class, as well as the cardinality of the dataset composed solely by enhancers and promoters (rows “Total E+P”) for genome version hg19 (Top table) and genome version hg38 (Bottom table)

Genome version	Labels	HepG2	K562	GM12878	Total	HelaS3
hg19	Active enhancer (AE)	1465	894	2878	*5237*	1847
	Inactive enhancer (IE)	34,556	34,392	28,156	*97,104*	32,179
	Active promoter (AP)	11,467	10,076	10,816	*32,359*	10,759
	Inactive promoter (IP)	96,184	82,829	73,891	*252,904*	79,004
	Total E + P	*143,672*	*128,191*	*115,741*	*387,604*	*123,789*
	Active exon (AX)	9931	9033	8226	9123	
	Inactive exon (IX)	19,071	20,261	19,078	22,071	
	Unknown (UK)	79,417	78,081	80,004	81,502	
	Total	*25,209*	*235,566*	*223,049*	*236,485*	
hg38	Active enhancer (AE)	7177	5524	11,589	*24,290*	
	Inactive enhancer (IE)	56,108	57761	51,696	*165,565*	
	Active promoter (AP)	14,092	12,524	14,036	*40,652*	
	Inactive promoter (IP)	85,789	87,357	85,845	*258,991*	
	Total E + P	*163,166*	*163,166*	*163,166*	*489,498*	

Column “Total” allows comparing the total cardinality of CRRs across the hg19 and the hg38-datasets. Since we also have non-CRRs regions for genome version hg19, row “Total” in the top table reports the total number of samples per cell line in the hg19 dataset

**Table 2:** Top Table: Unbalanced setup (first to fifth column): for each dataset and each task (first to fifth row from top to bottom) executed on data from genome version hg19, we report the class unbalancing ratio, computed as the ratio between the cardinality of the most-represented class and the cardinality of the less-represented class. Bottom table: the unbalancing ratios describing the unbalancing for data in hg38 are shown

Genome version	Task	Unbalancing ratios for different setups					
		Unbalanced setup					Full-balanced setup [34]
		HepG2	HelaS3	K562	GM12878	Average	All cell lines
hg19	IE versus IP	2.78	2.46	2.41	2.62	2.57	1
	AP versus IP	8.39	7.34	8.22	6.83	7.70	2
	AE versus IE	23.59	17.42	38.47	9.78	22.32	2
	AE versus AP	7.83	5.83	11.27	3.76	7.17	1
	AE + AP versus else	18.49	17.76	20.47	15.29	18.00	8
	Avg per cell line	12.22	10.16	16.17	7.66	* 11.55*	*2.8*

		Unbalanced setup					
		HepG2	K562	GM12878	Average		
hg38	IE versus IP	1.53	1.51	1.66	1.57		
	AP versus IP	6.09	6.98	6.12	6.39		
	AE versus IE	7.82	10.46	4.46	7.58		
	AE versus AP	1.96	2.27	1.21	1.81		
	Avg per cell line	4.35	5.30	3.36	4.34		

The fifth column (Average) shows the average unbalancing ratio over all the four cell lines, when the unbalanced setup is used. Task AE versus IE and task AE + AP versus else are, on average, the most unbalanced. Full-balanced setup (sixth column): the unbalancing ratio in each task is equal for all the cell lines. The comparison between the averages (over each cell lines) of the unbalancing factors (fifth and sixth columns) shows the striking difference between the two unbalancing modes.

**Fig. 1:**
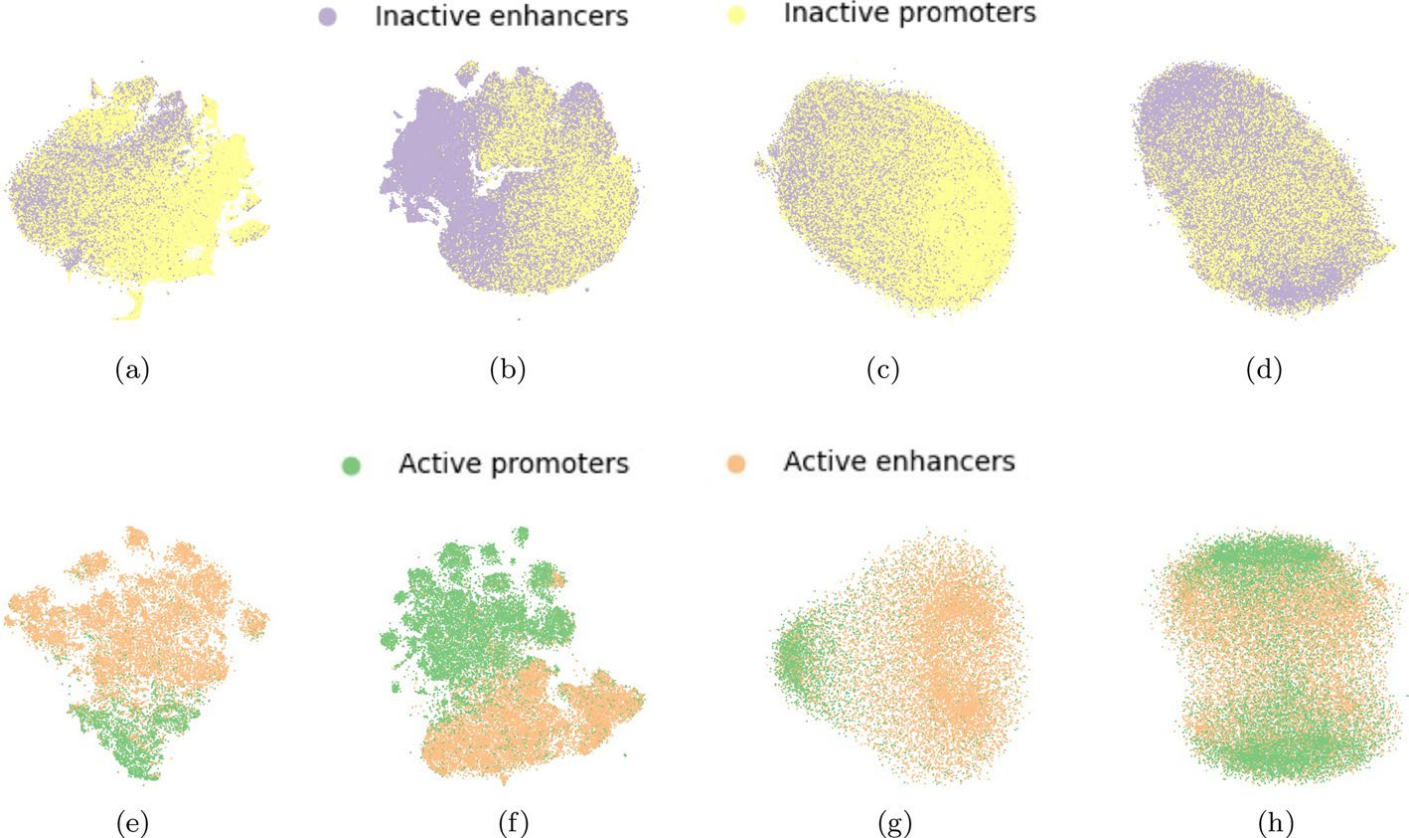
Top row: t-SNE projections of task IE versus IP: epigenomic data (**a**, **b**) and sequence data (**c**, **d**) for, respectively, hg19 dataset (**a**, **c**) and hg38-dataset (**b**, **d**) on one hold-out of the GM12878 cell line. Bottom row: t-SNE projections of task AE versus AP: epigenomic data (**e**, **f**) and sequence data (**g**, **h**) for, respectively, hg19 dataset (**e**, **g**) and hg38-dataset (**f**, **h**) on one hold-out of the GM12878 cell line

**Fig. 2:**
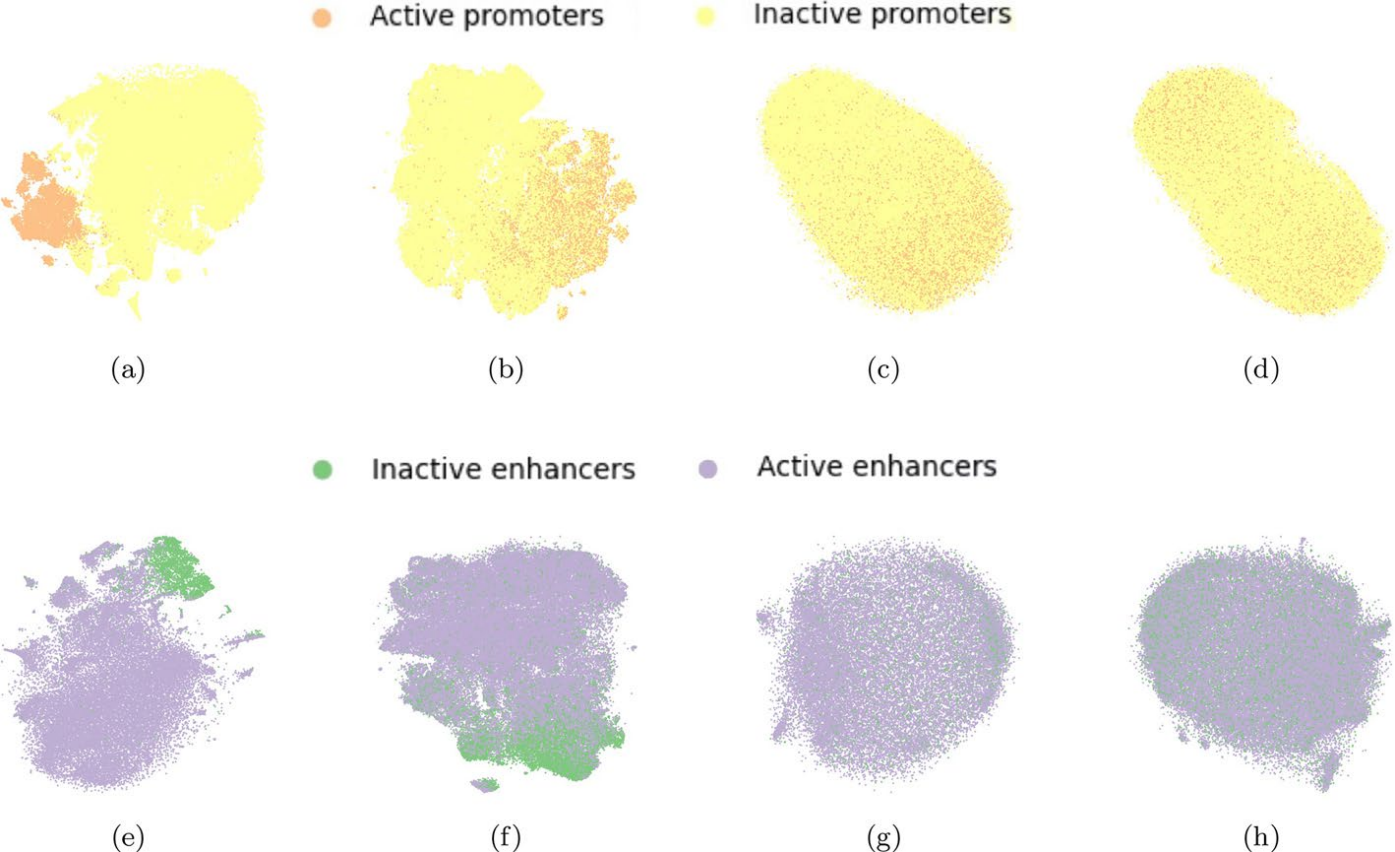
Top row: t-SNE projections of task AP versus IP: epigenomic data (**a**, **b**) and sequence data (**c**, **d**) for hg19 dataset (**a**, **c**) and hg38-dataset (**b**, **d**) on one hold-out of the GM12878 cell line, respectively. Bottom row: t-SNE projections of task AE versus IE: epigenomic data (**e**, **f**) and sequence data (**g**, **h**) for hg19 dataset (**e**, **g**) and hg38-dataset (**f**, **h**) on one hold-out of the GM12878 cell line, respectively

**Fig. 3:**
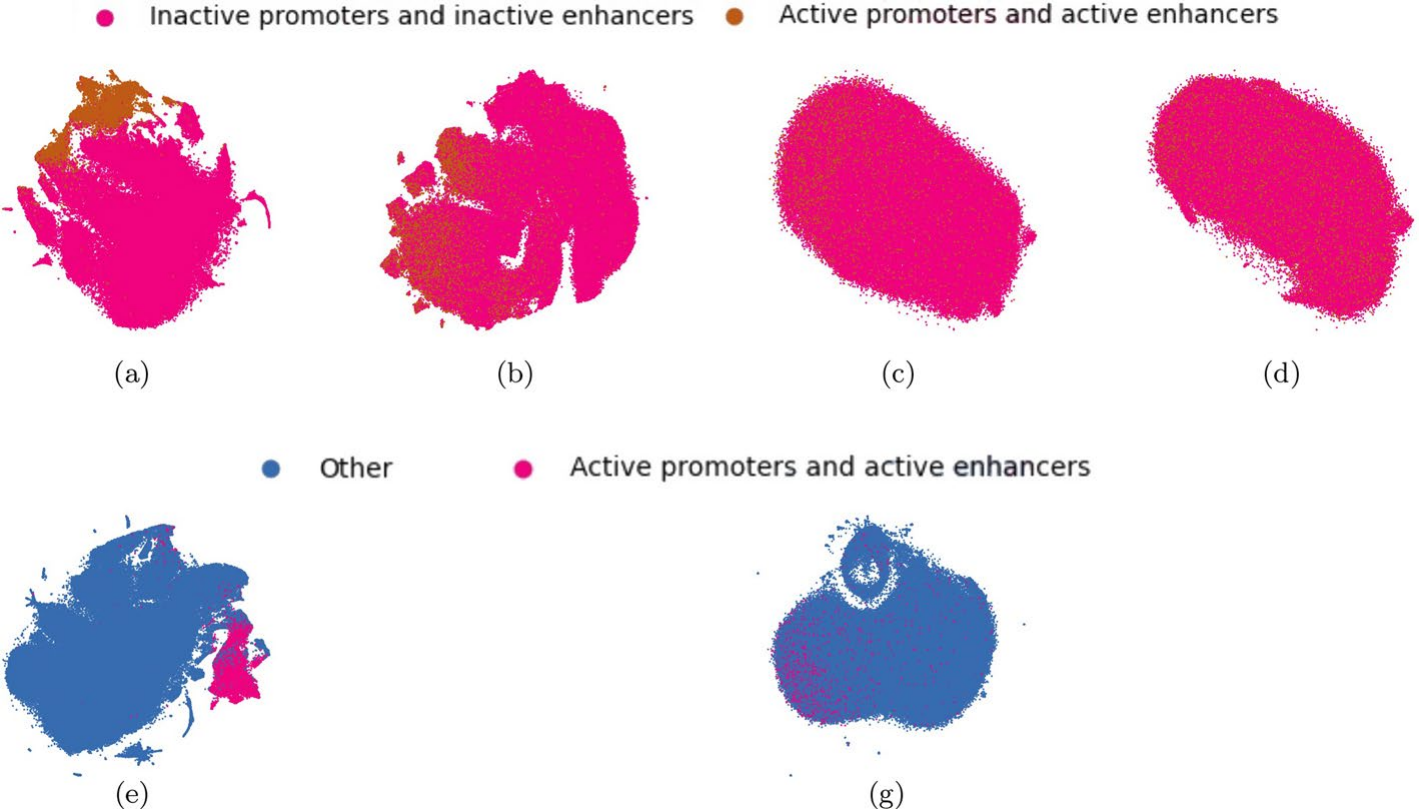
Top row: t-SNE projections of task AE + AP versus IE + IP: epigenomic data (**a**, **b**) and sequence data (**c**, **d**) for, respectively hg19 dataset (**a**, **c**) and hg38-dataset (**b**, **d**) on one hold-out of the GM12878 cell line. Bottom row: t-SNE projections of task AE + AP versus ELSE: epigenomic data (**e**) and sequence data (**g**) shown only for hg19 dataset

To further gain insights into the separability of the data in the different prediction tasks and in the two genome versions, we projected the test sets for both the FFNN models (epigenomic features) and CNN models (sequence data), and for all the cell lines and genome versions, on the first two components of the lower dimensional space computed by t-SNE [64].^1^ As an example, the projections of the test matrices with holdout from the cell line GM12878 are shown in Figs. 1,  2, and  3 for genome version hg19 (a, c, e, g) and genome version hg38 (b, d, f, h), respectively. Comparing all the t-SNE projections of epigenomic data (a, b, e, f) to those of sequence data (c, d, g, h), epigenomic data always shows a higher separability than sequence data. For hg19-data, the highest class overlap is visible for both epigenomic and sequence data in the task IE versus IP (see Fig. 1a, c), suggesting that this task will be the most difficult for both FFNN and CNN models. On the other side, the largest class separability (for both epigenomic and sequence data) is observed on hg38-data for tasks involving the separation of promoters from enhancers (IE versus IP in Fig. 1b, d, and AE versus AP in Fig. 1f, h). The other tasks involving the separation of active from non-active regulatory regions (AP versus IP—Fig. 2-b, d, and AE versus IE—Fig. 2f, h, and AE + AP versus IE + IP in Fig. 3b, d) show a higher class overlap with respect to data for hg19, for both epigenomic and sequence data.

### Bayesian optimization improves prediction of active regulatory regions

**Fig. 4:**
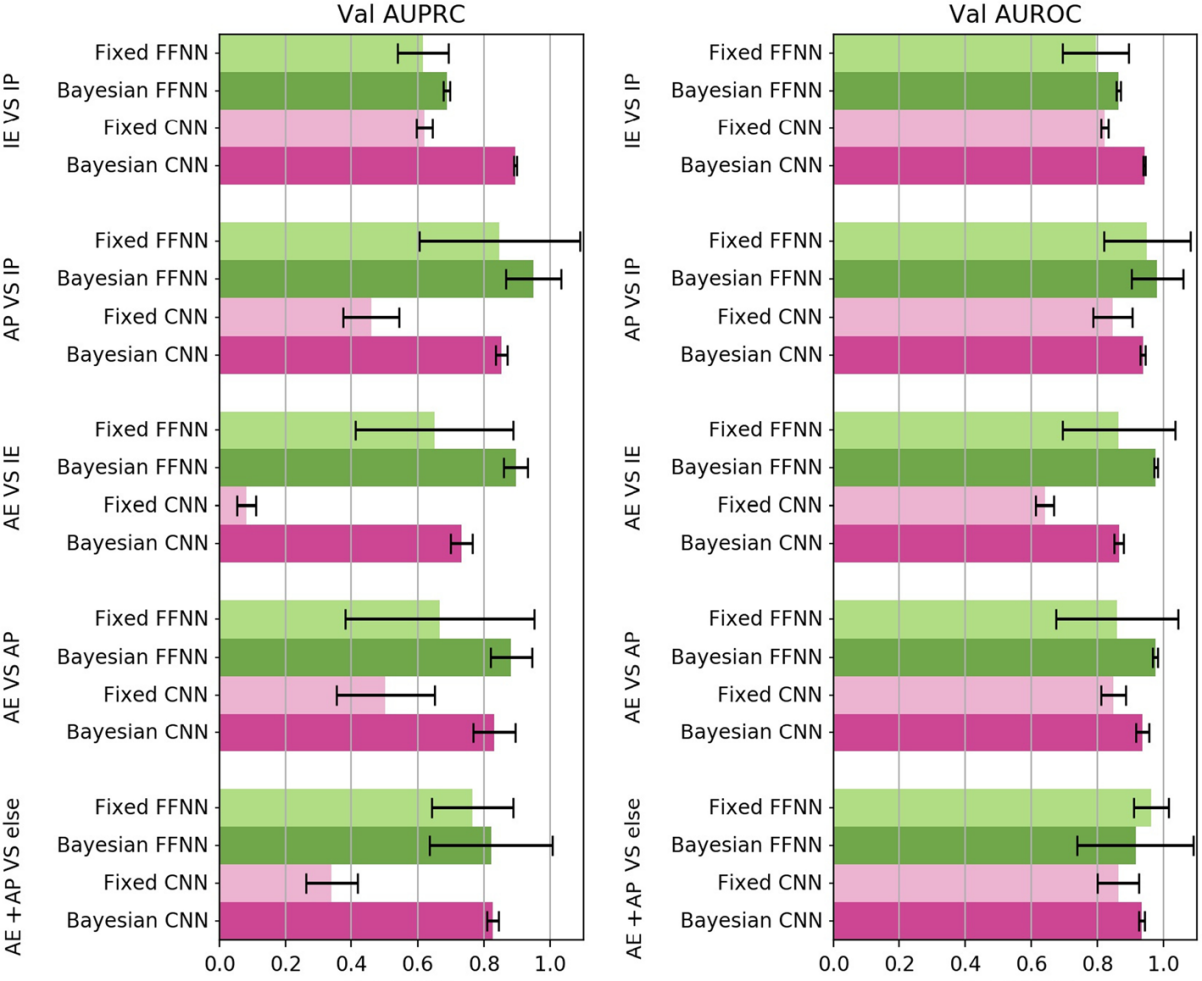
Comparison between fixed learning models and Bayesian models on data for genome version hg19. The plotted AUPRC (left) and AUROC (right) values are averaged over all the four cell lines and the multiple holdouts. Black bars represent standard deviations

Our first goal was to investigate the effect of model selection on FFNN and CNN models’ generalization performance and inspect whether a systematic exploration of the hyperparameter space leads to better classification results. To this end, we compared the fixed FFNN model, *fixed-FFNN*, to the optimized FFNN model, *Bayesian-FFNN*, and, similarly the fixed CNN model, *fixed-CNN* , to the optimized CNN, *Bayesian-CNN*, by performing the five classification tasks reported in “Experimental setup” section and [34] on the hg19 dataset (Tables 1, 2). For each of the five classification tasks, and each of the four models (*fixed-FFNN* versus *Bayesian-FFNN* and *Bayesian-CNN* versus *Bayesian-FFNN*), the mean AUPRC and AUROC computed over the testing holdouts (see “Experimental setup” section) and over the cell lines are shown, respectively, in the left and in the right Fig. 4. Paired Wilcoxon rank-signed test (at the 0.01 significance level) [61–63] was applied to detect statistically significant differences between the distributions of the AUPRC and AUROC values obtained by the fixed and optimized models.

Concerning FFNN, the *Bayesian-FFNN* outperforms the *fixed-FFNN*  in all the considered tasks and cell lines, achieving a statistically significant difference for the AUPRC metric (Wilcoxon test, $p-value < 0.01$). Similarly, also the *Bayesian-CNN* outperforms the *fixed-CNN*, achieving a statistically significant difference for the AUPRC metric (Wilcoxon test, $p-value < 0.01$).

Likewise, for the AUROC metric performance, the *Bayesian-FFNN* and *Bayesian-CNN* consistently outperform their fixed counterpart (Wilcoxon test, $p-value < 0.01$), except for the “AE + AP versus else” task, where the *fixed-FFNN*  achieves performance statistically indistinguishable from the *Bayesian-FFNN*. The overall results show that model selection through Bayesian optimization boosts both AUPRC and AUROC performance in most of the considered tasks.

### CNN models achieve performance close to FFNN models

When comparing the performance of the *Bayesian-FFNN* models to those of the *Bayesian-CNN* models, Wilcoxon test confirmed the superiority of AUPRC and AUROC achieved by *Bayesian-CNN* models in one task (IE versus IP), on two tasks (AE versus AP, AE + AP versus ELSE for AUPRC, and AP versus IP, AE + AP versus ELSE for AUROC) the two models showed not statistically significant differences, while in the other two tasks (AP versus IP, AE versus IE for AUPRC; AE versus IE, AE versus AP for AUROC) the *Bayesian-FFNN* models showed a better performance.

### Bayesian CNN is competitive with DeepEnhancer

Interestingly, *Bayesian-CNN* model working on sequence data showed promising results. To validate its effectiveness in CRR-activity prediction, we compared them to our implementation of the best performing DeepEnhancer model (the 4conv2pool4norm net, see “DeepEnhancer” section). In [33] the authors state that, though the DeepEnhancer networks have been developed for recognizing enhancers against background genome, they may be used for similar tasks; thus we tested *Bayesian-CNN* and DeepEnhancer (4conv2pool4norm) by using the four cell lines for hg19 to perform only the three classification tasks directly involving enhancers (IE versus IP, AE versus IE, and AE versus AP).

**Fig. 5:**
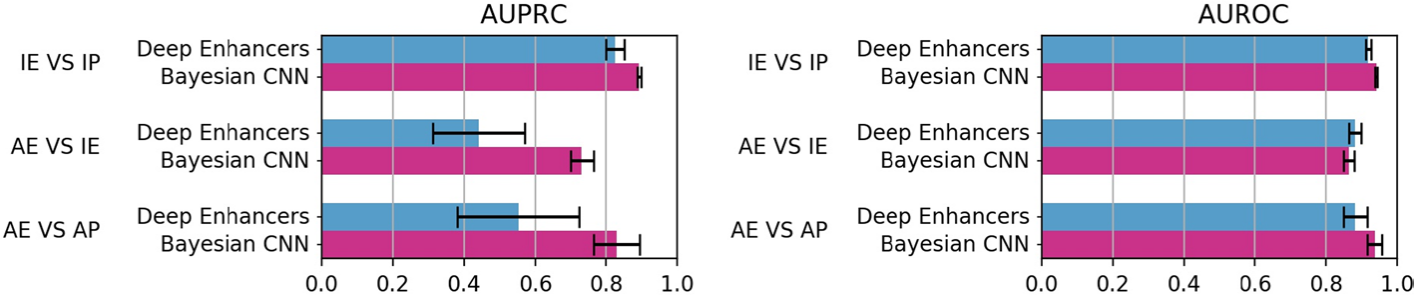
Comparison between *Bayesian-CNN* and DeepEnanhcer models on data for genome version hg19. AUPRC (left) and AUROC (right) values are averaged over the 10 multiple holdouts and the four cell lines. Black bars represent standard deviations

Both the models were assessed by using 10 holdouts over all the 4 datasets for hg19. Figure 5 shows, for each of the three tasks, the mean AUPRC (left) and the mean AUROC (right). One-sided Wilcoxon test confirmed that the differences visible in Fig. 5, for both AUPRC and AUROC, are statistically significant. Such performance further supports the results from the first experiment (“Bayesian optimization improves prediction of active regulatory regions" section), showing that model selection by Bayesian optimization allows to outperform state-of-the-art models. Further, these results show that a CNN model trained on genomic sequence alone may achieve an accurate classification performance for CRR activity prediction.

### Effect of different dataset-balancing setups

To solve the class unbalance problem, Li et al. [34] implemented a **fully-balanced setup**, where the construction of the training and test set (with train/test ratio 80%/20%) is followed by an under-sampling step of both the train and test splits, to decrease the effect of class unbalancing. In particular, each class in the training and test set must have no more than $3000$ examples, while a specific proportion among classes is enforced according to these ratios: (AE:AP:AX:IE:IP:IX:UK=1:1:1:2:2:1:10). Note that, in this way the class imbalance reported in the top of Table 2 is significantly reduced.

To understand the effect of such peculiar re-balancing, in this Section we trained and tested *Bayesian-FFNN* and *Bayesian-CNN* on the hg19 dataset by using the same experimental setting described for the previous experiments and by repeating all the prediction tasks under: (1) the aforementioned **fully-balanced setup**, (2) a **balanced setup**, where only the training set is balanced to have classes with equal cardinality (set to $3000$ samples here to obtain an objective comparison), and (3) the **unbalanced setup**, where no re-sampling is performed to maintain the original class distribution.

**Table 3:** For each balancing (columns) and task (rows), we report the average AUPRCs (top table) and average AUROCs (bottom table) obtained by the two Bayesian classifiers (the average is computed over the four cell lines)

Task	Balanced	Full-balanced	Unbalanced	Wilcoxon
**AUPRC**				
IE versus IP	0.627	0.787*	0.791*	0.251
AP versus IP	*0.745*	0.884*	0.901*	0.066
AE versus IE	*0.660*	**0**.**885**	0.814	
AE versus AP	*0.834*	**0**.**945**	0.856	
AE + AP versus else	*0.671*	**0**.**882**	0.824	
All tasks	*0.707*	**0**.**877**	0.837	
**AUROC**				
IE versus IP	0.82*	0.819*	**0**.**903**	0.046
AP versus IP	0.919	0.931	**0**.**960**	
AE versus IE	0.893*	**0**.**921**	0.9205*	0.052
AE versus AP	–	0.960*	0.956*	0.249
	0.952*	–	0.956*	0.035
AE + AP versus else	0.929*	**0**.**956**	0.925*	0.066
All tasks	0.903	0.917	**0**.**933**	

Character * marks not statistically different pairs and, in this case, the last column reports the computed *p* value > 0.01. Bold text highlight the best performance, when this is statistically different from all the other values

**Fig. 6:**
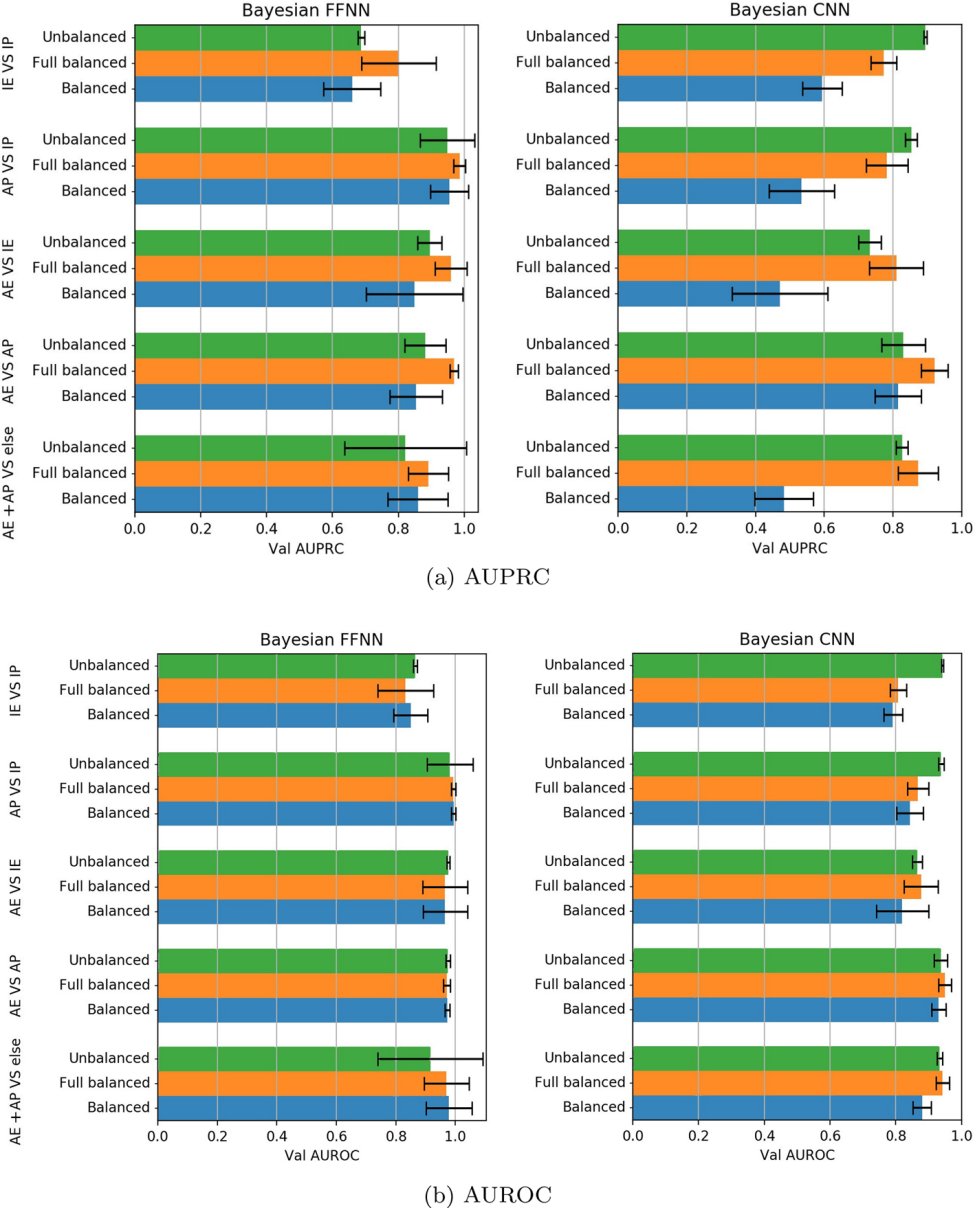
Comparison between different balancing set-ups on data for genome version hg19. The AUPRC (top) and mean AUROC (bottom) values obtained by *Bayesian-FFNN* (right) and *Bayesian-CNN* (left) when the three balancing set-ups are used are averaged over the four cell lines and the ten holdouts. Black bars represent standard deviation

For a detailed comparison of the achieved performance, the top (bottom) of Table 3 reports the average (over all the cell lines and the two Bayesian FFNN and CNN methods) of the AUPRC (AUROC) values achieved for each task (on rows) by using different balancing modes (on columns). In the last row the average AUPRC (AUROC) values over all the tasks are reported for each balancing set-up. The statistical significance of the difference in the average AUPRC (and AUROC) values between different balancing set-ups was assessed by the Wilcoxon rank-signed test with $p<0.01$. In the last column of Table 3, we report the Wilcoxon *p* value computed when comparing AUPRCs where the difference is not statistically significant. Precisely, for each task in Table 3, character * marks not significantly different values (AUPRC or AUROC) according to a Wilcoxon test, while bold text highlights the highest AUPRC (AUROC). Figure 6 summarizes the comparison between the different balancing set-ups.

**Fig. 7:**
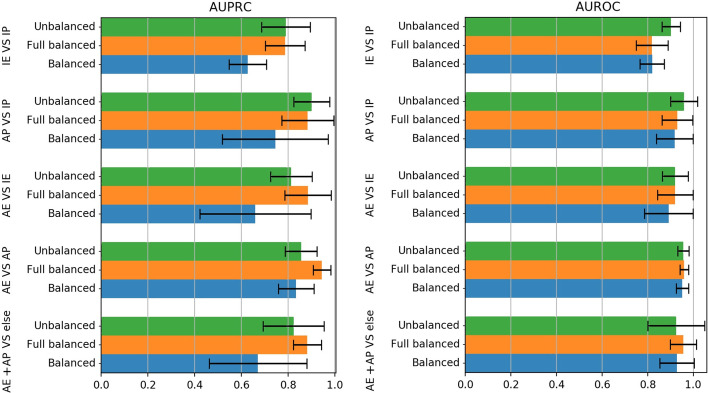
Comparison between the three balancing setup on hg19 dataset. The barplot on the left shows the mean AUPRC averaged over the Bayesian models (*Bayesian-FFNN* and *Bayesian-CNN*), the four cell lines, and the ten holdouts. On the right, mean AUROC is averaged over the Bayesian models, cell lines, and holdouts. Black bars represent standard deviations

Observing the AUPRCs in Table 3 (see also Fig. 7), we note that the balanced experimental set-up is the one obtaining the worst performance in all the tasks; a similar behaviour is visible in the AUROCs (bottom table), though in this case the differences are often not statistically significant. On the AUPRC, the Wilcoxon test confirms that, on average, the full-balanced setup produces higher AUPRC scores (AE versus IE, AE versus AP, AE + AP versus ELSE) or, anyway, scores that are comparable to the best performing setup (IE versus IP and AP versus IP).

### Bayesian optimization also improves performance on hg38-dataset

**Fig. 8:**
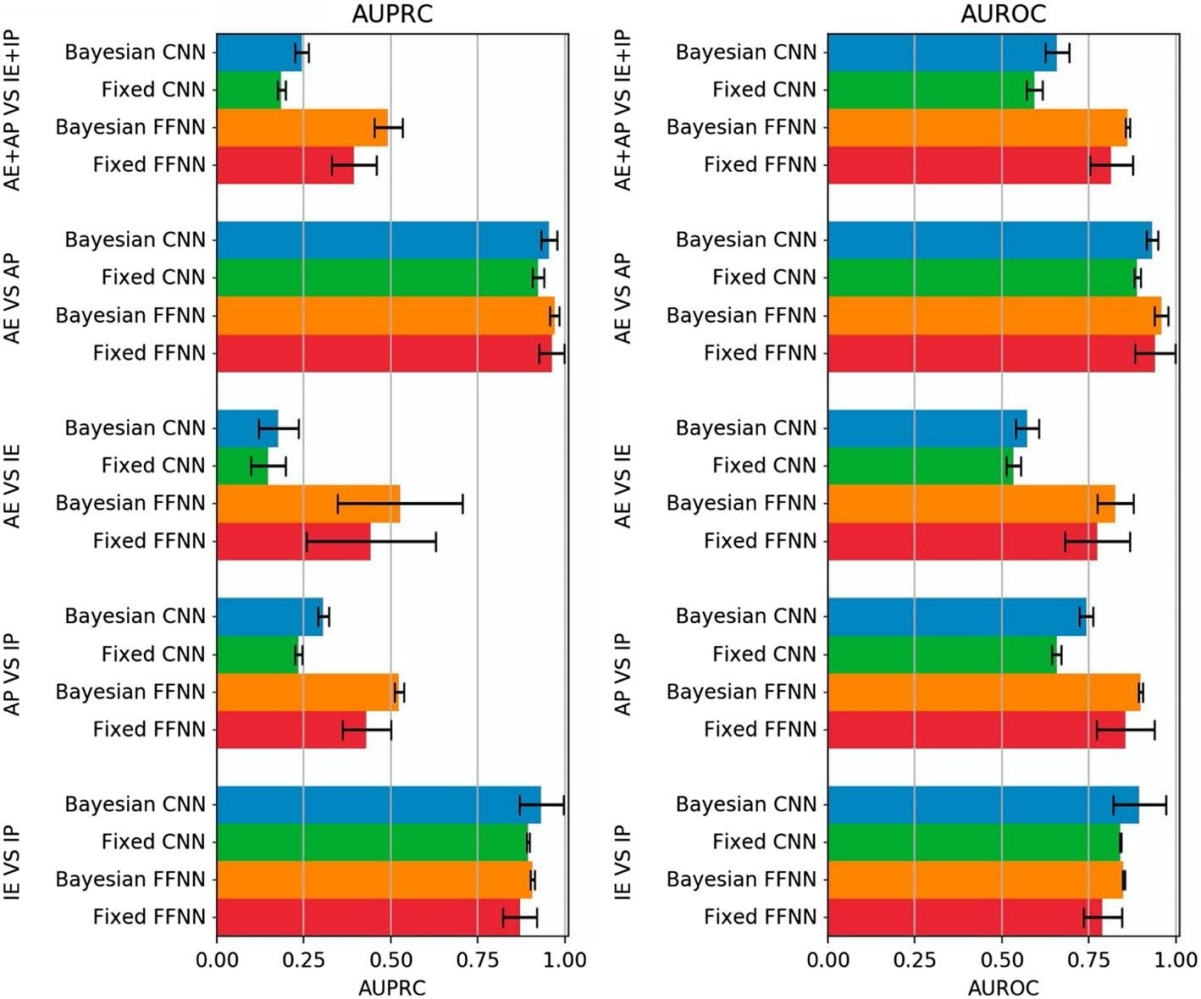
Comparison between fixed and Bayesian models on the data for genome version hg38. AUPRC values (left) and AUROC values (right) are averaged over the 10 multiple holdouts and the three available cell lines. Black bars represent standard deviations

Figure 8 compares the AUPRC and AUROC performance of the Bayesian models (*Bayesian-FFNN* and *Bayesian-CNN*) with their fixed counterparts (*fixed-FFNN* and *fixed-CNN*), averaged over $20$ holdouts, trained and evaluated on the hg38 dataset. The Wilcoxon signed-rank test ($p< 0.01$) confirmed that the Bayesian models always outperform their fixed counterparts in both the AUPRC and AUPRC metrics, in all the considered tasks and cell lines, confirming the results obtained with hg19 data.

By comparing Figs. 8 and 4, we observe that we obtain better results with the hg38-dataset than those achieved with hg19 data when we classify promoters versus enhancers (i.e. IE versus IP and AE versus AP tasks), but worse results when we classify active versus inactive CRRs (i.e. AP versus IP, AE versus IE and AE + AP versus ELSE).

The optimal *Bayesian-FFNN* architectures chosen for the task AE versus IE for cell line GM12878, for both the hg19 and hg38 datasets, are visualized, respectively in Fig. 10a, c. The two models look similar in their pyramidal shape (the number of units in each dense layer decreases from input to output); however, as noted in section *fixed-FFNN and Bayesian-FFNN*, while the pyramidal shape is constrained in the *Bayesian-FFNN* meta-model developed for the hg19 dataset, that for the hg38 dataset could allow building both pyramidal and rectangular architectures.

The optimal *Bayesian-CNN* architectures selected for the task AE versus IE for cell line GM12878, for both the hg19 and hg38 human genome assemblies, are visualized in, respectively, Fig. 10b, d.

## Discussion

The present work aims at providing further knowledge in the field of CRR activity prediction. More precisely, the experiments presented in “Results” section have been designed to: (1) compare the performance obtained by using FFNN models processing epigenomic features to that obtained by CNN models working on sequences; (2) understand whether model tuning can improve the prediction performance; (3) provide insights about the different rebalancing procedures exploited at the state-of-the-art to handle the data imbalance issue; (4) validate the results obtained with the two genome datasets: the first one based on the hg19/GRCh37 genome version while the second on the hg38/GRCh38 version.

In particular, results reported in “CNN models achieve performance close to FFNN models” section show that the analysis of sequence data through CNN leads to results comparable to those obtained with FFNN and epigenomic features, and suggests that a multimodal approach integrating the information carried by both the data types could achieve increased performance.

Further, experimental results achieved on both the hg19 and hg38 datasets show that a proper choice of the model hyper-parameters through Bayesian optimization allows improving the model generalization capability by systematically increasing the performance of non optimized fixed models.

The different results obtained with hg19 and hg38 data sets can be explained by their different distributions. Indeed t-SNE plots reveal that active versus inactive CRRs (Figs. 2, 3) in hg38 data show larger overlaps than in hg19 data. This can be in turn explained by the fact that hg38 on the one hand includes more CRR samples and on the other hand the FANTOM5 labeling (active versus inactive) significantly changed between the two human genome assemblies (see “Datasets” section for more details).

Moreover, with the hg38-dataset, differently from the hg19 dataset, the *Bayesian-FFNN* approximately doubles the AUPRC performance of the *Bayesian-CNN* in the active versus inactive tasks (Figs. 4, 8). This behaviour is expected, since it is well-known that epigenetic data are more informative than sequence data in distinguishing AP versus IP or AE versus IE.

**Fig. 9:**
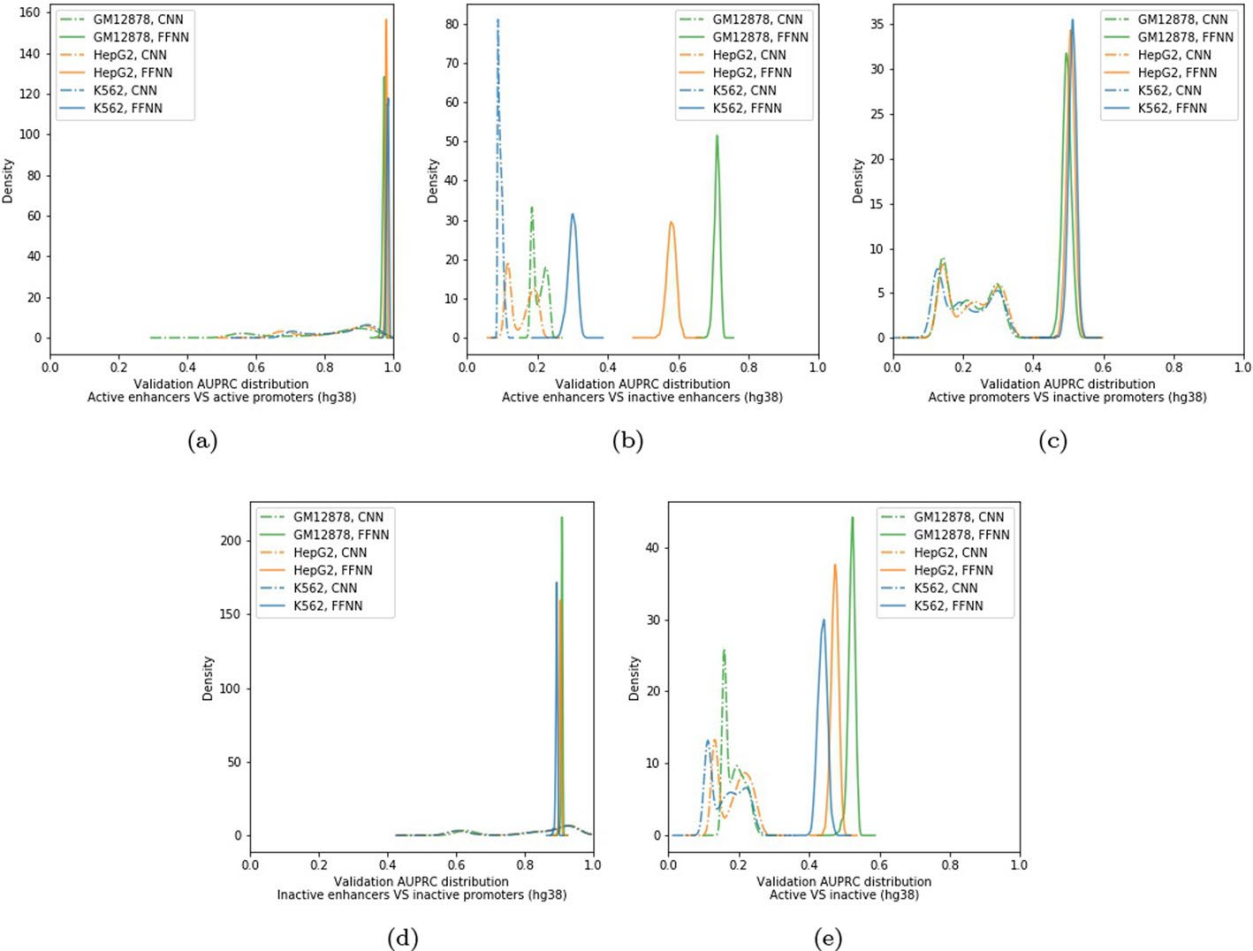
Distributions of the Validation AUPRC sampled during Bayesian optimization of the *Bayesian-FFNN* meta-models (continuous lines) and *Bayesian-CNN* meta-models (dashed lines), when trained on the hg38 dataset. Different colors correspond to different cell lines. **a** Active Enhancers versus Active Promoters (AE versus AP); **b** Active Enhancers versus Inactive Enhancers (AE versus IE); **c** Active Promoters versus Inactive Promoters (AP versus IP); **d** Inactive Enhancers versus Inactive Promoters (IE versus IP); **e** Active Enhancers and Promoters versus Inactive Enhancers and Promoters (AE + AP versus IE + IP)

Figure 9 shows the distribution of the validation AUPRC values achieved by the FFNN and CNN models generated during the Bayesian optimization process for the different considered tasks and cell lines. The performance of the different deep neural network models can quite largely vary dependently of the choice of the hyperparameters, thus confirming that Bayesian optimization is crucial to improve performance. Moreover, observing the obtained plots, we may note that the *Bayesian-FFNN* distributions (Fig. 9, continuous line) have a unique local maxima, while those computed while optimizing the *Bayesian-CNN* meta-model (Fig. 9, dashed line) contain up to three local maxima. This may partially motivate the slower convergence we observed when optimizing the *Bayesian-CNN* meta-model. On the other side, *Bayesian-FFNN* generally rapidly reaches convergence to a local maxima.

**Fig. 10:**
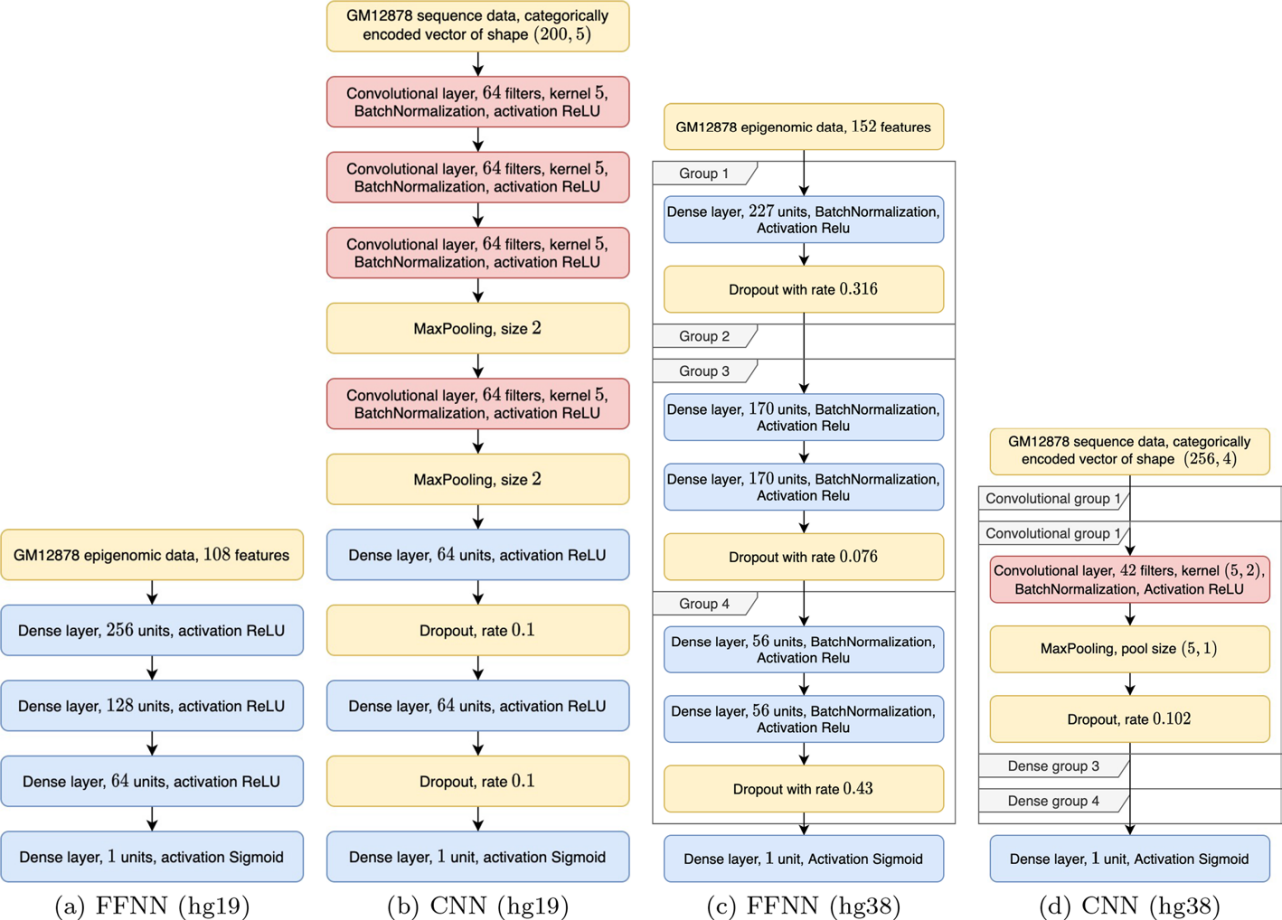
Models selected by Bayesian optimization for the AE versus IE task, cell line GM12878, for genome assembly hg19: **a** FFNN; **b** CNN, and for genome assembly hg38: **c** FFNN; **d** CNN. Group 2 in **c** and groups 1, 3 and 4 in **d** are void since no hidden layers have been selected by the Bayesian optimization procedure

The best parameters selected by the Bayesian optimization procedure depend on the task and the considered cell line, which suggest the existence of different underlying structures. Examples of the *Bayesian-FFNN*  and *Bayesian-CNN*  architectures selected by Bayesian optimization for the task AE versus IE applied to cell line GM12878 on the hg19 and the hg38 datasets are shown in Fig. 10. Full results about the best selected hyperparameters for each task and cell line are available in the github site (see Availability of data and materials).

The potentials of Bayesian models trained on sequence data have also been confirmed by the comparison with DeepEnhancer, a state-of-the-art approach for active enhancer region prediction [33] which similarly leverages CNNs trained on sequence data.

It has to be finally pointed out that results are also strongly dependent on the chosen experimental setup. Indeed, crucial experimental choices, such as the dataset rebalancing technique, may positively bias the obtained results, therefore producing optimistic estimates of the model performances. In particular results reported in “Effect of different dataset-balancing setups” section show that (1) training set rebalancing should be carefully designed in order to avoid loosing discriminative information, which would result in decreased performance; (2) test set rebalancing should not be performed since it produces over-optimistic, unreliable results.

Regarding training set rebalancing, referring to Table 3 and Fig. 7, we believe that the low performance of the balanced setup may be due to the fact that the training set re-balancing is performed by sub-sampling, which discards a (sometimes) large amount of training samples. This reduces the information made available to the learner for training; as a result, the learner has difficulties in effectively learning the inter- and intra-class variability, therefore resulting in a reduced generalization capability. Though this reduction affects both balanced and full-balanced modes, in the latter case the test set is also reduced in size, hence minimizing the impact of misclassification. Moreover, since the unbalanced setup works on all the available training cases, the learner sees all the training set variability and this may be the reason why the unbalanced mode scores sometimes better than the full-balanced, and always better than the balanced mode (Figs. 6, 7).

Test set balancing may be responsible for the high scores registered by the fully-balanced setup; hence, we believe that more realistic results are obtained by a complete unbalanced setup, since in a real-world setting there is no way to balance the test set, as labels to be predicted are not known.

## Conclusions

In this work we presented experiments aimed at investigating the usage of deep neural network models for predicting CRR activity.

In particular, we implemented a FFNN, which works on CRRs annotated with epigenomic features and conservation scores, and a CNN, which processes the same CRRs coded by their genomic sequence.

Given such setting, we firstly showed that model selection through Bayesian optimization has the potential for improving the classification results computed by both architectures. This result was proved on the CRRs dataset provided by the authors of [34], and validated on epigenomic and sequence data from genome version hg38/GRCh38. Bayesian optimization is also fundamental to improve CNN results, as shown by our experimental comparison of *Bayesian-CNN* with the current state of the art DeepEnhancer model [33]. To the best of our knowledge, this is the first time that the Bayesian optimization approach is used to tune deep learning models to predict CRRs.

To analyze the effect of the dataset balancing performed in the DECRES [34] work, we experimented different rebalancing techniques, showing that balancing the test set may lead to an over-optimistic estimation of the generalization performances of the model. From the experimental results, we infer that balancing set-ups must be carefully designed to avoid incurring misleading model evaluations due to biases induced in the data distributions.

Results obtained with hg38 show that the task of predicting active versus inactive regions, for both promoters and enhancers, is still an open problem. We obtained an average AUPRC greater than 0.5 and an AUROC grater than 0.8 for the AE versus IE task, showing that there is room to further improve performance.

Since the promising results achieved with genomic sequences suggest that also this data type carries salient information, we plan to develop multimodal architectures where two specialized neural branches, a *Bayesian-FFNN* branch and a *Bayesian-CNN* branch, will separately extract the information from, respectively, epigenomic and sequence data, and their resulting embedded data representations will be integrated at a higher level by a merging fully connected neural module producing the final output.

## Methods

To detect active CRRs using either epigenomic data or genomic sequences we developed two (deep [65, 66]) neural network models: FFNN models are applied to one dimensional vectors containing epigenomic features, while CNN models are applied to process a two-dimensional sparse vector representing the one-hot-encoded genomic sequence data.

In particular FFNN models [36] are a class of weighted acyclic graphs composed of layers of neurons (an *input layer*, a number of *hidden layers*, and an *output layer*) interconnected by weighted edges, where the weight of each edge is learnt during the training phase.

FFNN operate by feed-forward propagating the signals from the input layer, to all the consecutive hidden layers, and then to the output layer. In more detail, while the *input layer*, is used to ingest the input vectors and diffuse them to the next hidden layer, each neuron in the generic hidden layer performs a *weighted sum* of the signals it receives from the preceding layer, where the weights are those relative to the edges transporting the signal itself. The weighted sum is then input to an *activation function* (e.g. ReLU, sigmoid, tanh [67]), which computes the neuron activation by normalizing the computed value and introducing non-linearity. In the *output layer* each neuron is associated to a prediction (class) and its activation defines the neuron response for that prediction.

The aforementioned structure (*architecture*) of FFNN models is characterized by (architectural) *hyperparameters* that define the network depth (number of hidden layers) and its extent (the number of neurons for each layer). In neural networks, the model parameters (i.e. the weights) are automatically learnt from the data through the backpropagation algorithm, while all the other parameters, i.e. the number of hidden layers and the number of hidden neurons, or the learning rate, that are not directly learnt during backprogation are usually called model hyperparameters.

CNNs [36] are neural networks that use convolutions instead of full vector and matrix multiplication as in FFNNs, and are designed to work on $n$-dimensional signals, e.g. images, where the relationship between neighboring elements must be accounted for.

CNNs essentially apply consecutive filtering operations to the input signals and their strength is due to their ability to automatically infer the optimal filter weights, that is the weights maximizing performance on the training data. In the context of DNA sequence processing this means that CNNs are able to identify motifs in a fully automated way, thus allowing to find binding sites of transcription factors that regulate the expression of genes. More details on CNNs are available, e.g. in [36].

Both models basically apply the backpropagation algorithm to learn the weights, in order to optimize an objective function that in classification problems is typically represented by Binarized or Multi-class cross-entropy [68]). To this aim, several optimizing algorithm may be chosen (e.g. Stochastic Gradient Descent—SGD, Root Mean Square Propagation—RMSProp, Adaptive Moment Estimation—Adam, Nesterov-accelerated Adaptive Moment Estimation—Nadam, and many others [69]), which are guided by a set of hyperparameter values (e.g. learning rate, momentum, batch-size, maximum number of epochs, early stopping patience), which are often manually set to suggested default values or are set based on previous experience.

In this work, the Nadam optimizer [70] is used to find the weight values optimizing the binary cross-entropy (BCE) loss over the training set, which is computed as:

$$BCE(y_{i}, {\hat{y}}_{i}) = -(y \log ({\hat{y}}_{i}) + (1 - y) \log (1 - {\hat{y}}_{i})) $$

where $y_i$ is the true label for sample *i* and ${\hat{y}}_i$ is the predicted label.

The aforementioned description clarifies that the design and development of deep neural network models is not trivial, since a high-number of (architectural and training) hyperparameters must be properly set.

In this section we describe the models we developed in order to assess the usage of Bayesian optimization (“Bayesian optimization” section) in the model selection phase for the task of CRR activity classification.

In particular we firstly developed FFNN and CNN models defined by fixed hyperparameters, whose values are chosen according to previous state-of-the-art studies [34], which will be referred to as “fixed” models. Starting from the fixed models, we developed “Bayesian” models whose “optimized” hyperparameter values are chosen by Bayesian optimization in a search space that includes the points corresponding to the hyperparameters of the fixed FFNNs. In this way, we obtained two “fixed” models, i.e. *fixed-FFNN* and *fixed-CNN*, and two “Bayesian” models, i.e. *Bayesian-FFNN* and *Bayesian-CNN*, fully described in Sections *fixed-FFNN and Bayesian-FFNN* and *fixed-CNN and Bayesian-CNN*.

While the *fixed-FFNN* models have been developed based on previous research works [34] and may be used as baseline models for comparison with their Bayesian version, to provide an exhaustive evaluation of CNN models trained on raw sequence data we exploited the best performing DeepEnhancer model, described in “DeepEnhancer” section.

All the models were developed using Keras [71] with TensorFlow backend [72].

### Bayesian Optimization

The idea behind Bayesian optimization is that a function (the “objective function”), characterized by high cost for the evaluation of each point of a bounded domain, can be approximated by building a probabilistic model (the “Surrogate function”) which is cheaper to query. Optimization can then be performed by substituting the objective with the surrogate function providing at the same time a possible minimum or maximum for the latter.

As the surrogate function represents an “a priori” distribution of the objective function, given some observation points obtained by evaluation of the objective, it is possible to exploit Bayes’s rule to generate an “a posteriori” estimation of the (objective) function and then update the probabilistic model (surrogate function). Candidates of observation points are suggested through an appropriate “Acquisition function” which uses the information gained by the probabilistic model (estimated by the already observed points) for suggesting the next candidate.

Depending on the task, different acquisition functions may be used, but their common trait is that they all act upon the criteria of “exploration versus exploitation”, so that the sequence of suggested points will provide a better overlook of the objective function (exploration) or a better identification of its maximum/minimum (exploitation). A comprehensive review of possible acquisition functions is available at [73].

We used the Python packages *Ray* [74] and *HyperOpt* [75] to implement Bayesian optimization in our deep neural network framework available at https://github.com/AnacletoLAB/meta_models.

### *fixed-FFNN* and *Bayesian-FFNN*

We initially addressed the prediction of active regulatory regions by developing a *fixed-FFNN* model, whose architecture and learning hyperparameters have been chosen by considering the work described in [34], where authors developed a successful neural network and applied grid search to automatically select the architecture and learning hyperparameters.

Precisely, in our *fixed-FFNN* (schematized in Table 4A) the neural network architecture is composed by cascading three fully-connected layers composed of 16, 4 and 2 neurons with ReLU [76] activation function, respectively. A final layer structured as a single neuron with sigmoid activation function acts as output layer, computing the final binary predictions. During network training, weight values were adjusted by Stochastic Gradient Descent technique with fixed learning rate of 0.5, learning rate decay of 0.1, $l_2$ regularizer of 0.0, no momentum and a maximum of 64 epochs. While the number of layers, the activation functions, and the optimization technique are those selected by grid search in [34], the values of the learning rate, the learning rate decay, and $l_2$ regularizer were chosen by performing a brief manual tuning of the learning hyperparameters, since in [34] the exact ratings for such parameters were not reported. Moreover, after considering both the suggestions reported in [34] and the research works reported in [77, 78], where authors observed that high ratings for batch-size decrease the generalization capability of the network, we chose a fixed and relatively small value for the batch size parameter (32).

Exploiting the knowledge derived from experiments performed with the *fixed-FFNN*, we initially performed experiments on the hg19 dataset by developing a *Bayesian-FFNN* whose strength lies in the automatic model selection through Bayesian optimization.

To attain a fair comparison with the deep neural networks proposed in [34], *Bayesian-FFNN* adopted a similar hyperparameter space (Table 4B). We substituted the computationally expensive grid search with Bayesian optimization [79], which maximizes the mean AUPRC computed over the validation sets of 10 internal holdouts (see “Experimental setup” section).

According to the experiments on the hg19 dataset, the architecture chosen by Bayesian optimization for the *Bayesian-FFNN* was often at the higher boundary of the search space. Indeed, the chosen models were often composed by three fully connected layers each composed by the maximum allowable number of units (256, 128, and 64). On the other side, the learning parameters were selected in the lower spectrum of the continuous search interval for all parameters but for the maximum number of epochs.

These results suggested to explore a larger hyperparameter space and to this end for the hg38 data, we decided to develop more complex *Bayesian-FFNN* models, which could allow exploring a wider search space (Table 4C).

In more detail, the novel meta-model is composed till to $n=4$ groups having each one from 0 to 3 hidden layers, where a value equal to 0 means that the layer will be removed. Each dense hidden layer has batch normalization and ReLU activation, its width is chosen by Bayesian optimization in the discrete range $\{0, 256\}$, and the considered layer is dropped if the optimal width equals 0. Finally a dropout layer is added to regularize the output of each group, and also the dropout rate is chosen (in the range $[0, 0.5]$) by Bayesian optimization; again, if the chosen rate value equals 0, the dropout layer is removed.

Note that the described *Bayesian-FFNN* meta-model architecture for the hg38 dataset allows to span a wide search space, which includes the search space of the *Bayesian-FFNN* meta-model for hg19 as a subset. In particular, while the hg19 meta-model constraints the final architecture to have a pyramidal shape, with decreasing number of neurons from the input to the output layer, the meta-model for hg38 may also allow to build a rectangular shape, where consecutive layers have the same width.

**Table 4:** FFNN hyperparameter space explored with hg19 and hg38 data through Bayesian optimization. A. Architecture and learning hyperparameters of the *fixed-FFNN*; B. Architecture and hyperparameter space of the *Bayesian-FFNN* models trained on the hg19 dataset; C. Architecture and hyperparameter space of the *Bayesian-FFNN* models models trained on the hg38 dataset

Layers	Units	Activation
**A: fixed-FFNN**		
Dense	16	ReLU
Dense	4	ReLU
Dense	2	ReLU
Output	1	Sigmoid
**Learning parameters**		
Learning rate	0.5	
Learning rate decay	0.1	
l2 regularizer	0.0	
Batch size	32	
Optimizer	SGD	
Max no. of epochs	64	

Layers	Hyperparameter space	Activation
**B: Bayesian-FFNN (hg19 dataset**)		
No. of dense layers	{0, 1, 2, 3}	
No. of units layer 1	{256, 128, 64, 32, 16, 8, 4, 2}	ReLU
No. of units layer 2	{128, 64, 32, 16, 8, 4, 2}	ReLU
No. of units layer 3	{64, 32, 16, 8, 4, 2}	ReLU
Output	1	Sigmoid
**Learning parameters**		
Learning rate	[0.1, 0.5]	
Learning rate decay	[0.01, 0.2]	
l2 regularizer	[0, 0.1]	
Batch size	[32, 256]	
Optimizer	SGD	
Max no. of epochs	[32, 1000]	
**C: Bayesian-FFNN (hg38 dataset**)		
Groups	$n=4$	
No. of hidden layers, composing the group	$\{0, \ldots , 3\}$	
No. of units of the dense layer	$\{0, \ldots , 256\}$	ReLU
Dropout	$[0, \ldots , 0.5]$	
Output	1	Sigmoid
**Learning parameters**		
Learning rate	[0.1, 0.5]	
Learning rate decay	[0.01, 0.2]	
l2 regularizer	[0, 0.1]	
Batch size	[32, 256]	
Optimizer	SGD	
Max no. of epochs	[32, 1000]	

In Tables B and C, for each otpimized hyperparameter, the search hyperparameter space is shown, where square brackets are used for continuous hyperparameter spaces, while curly brackets are used for discrete ones. “Dense” refers to fully connected layers

### *fixed-CNN* and *Bayesian-CNN*

Similar to the experiments run on FFNN, for CNN we experimented with the usage of a fixed architecture, to assess whether this approach may effectively recognize active regulatory regions, and an optimized architecture with hyperparameters chosen by Bayesian optimization, to discover whether Bayesian optimization may improve performance obtained by processing genomic sequences.

The *fixed-CNN* model is outlined in Table 5A. The core of the network is composed by three consecutive blocks, each composed by three (consecutive) convolutional layers followed by one 1-dimensional max/average pooling layer. The number of units in the three convolutional layers of each block, as well as the filter sizes, are fixed. A filter size of 5 for the first three convolutional layers was chosen as this represents a reasonable motif size [80]. All neurons in each layer have ReLU activation function with the exception of the output layer where the output neuron has sigmoid activation. The Nadam algorithm [70] adjusts the weight values, the learning rate is set to 0.002, and the batch size equals 256 examples.

Bayesian optimization was firstly experimented by using the hg19 dataset to perform model selection. Precisely, Bayesian optimization was used to choose the best architecture (number of convolutional groups from 1 to 3, and, for each layer, a number of filters lower than that of the *fixed-CNN* model) that maximizes the mean AUPRC computed over the validation sets of the 10 internal holdouts (“Experimental setup” section). The resulting *Bayesian-CNN* has a meta structure shown in the bottom of Table 5B. The meta-model uses the default Nadam learning learning rate (0.002) and batch size (256).

When running experiments on the hg38 dataset, in line with the experiments on the FFNN models, we re-designed the *Bayesian-CNN* meta-model to allow exploring a wider search-space. The novel meta-model and the wider hyperparameter space are schematized in Table 5C; the meta-model allows developing models with $n_{conv} \in \{0, 2\}$ convolutional blocks and $n_{dense} \in \{0, 2\}$ dense blocks, where both the values of $n_{conv}$ and $n_{dense}$ are chosen by Bayesian optimization. Each convolutional block is composed from 0 to 3 convolutional layers with batch normalization and ReLU activation, followed by a 2D max pooling layer and a dropout layer. The dense blocks are composed by a fully connected layer with batch normalization and ReLU activation, followed by a dropout layer. The hyperparameter space explored by the *Bayesian-CNN* is detailed in Table 5C.

**Table 5:** CNN hyperparameter space explored with hg19 and hg38 data through Bayesian optimization. A. Architecture and learning hyperparameters of the *fixed-CNN*; B. Architecture and hyperparameter space of the *Bayesian-CNN* models trained on the hg19 dataset; C. Architecture and hyperparameter space of the *Bayesian-CNN* models models trained on the hg38 dataset

Layers	Type	Units	Kernel	Activation	Notes
**A: fixed-CNN **					
3	Convolutional	64	5	ReLU	–
1	Max pooling 1D	–	–	–	size 2
3	Convolutional	128	3	ReLU	–
1	Max pooling 1D	–	–	–	size 2
3	Convolutional	128	3	ReLU	–
1	Average pooling 1D	–	–	–	–
1	Dropout	–	–	–	Probability 0.5
2	Dense	10	–	ReLU	–
1	Dropout	–	–	–	Probability 0.5
1	Dense	1	–	Sigmoid	–
**Learning parameters**					
Learning rate	0.002				
Batch size	256				
Optimizer	Nadam				
Epochs	100				

Layers	Type	Units	Kernel	Activation	Notes
		Hyperparameter space	Hyperparameter space		
**B: Bayesian-CNN (hg19 dataset)**					
3	Convolutional + batch norm	{32, 64, 128}	5	ReLU	–
1	Max pooling 1D	–	–	–	Size 2
1	Convolutional + batch norm	{32, 64, 128}	{5, 10}	ReLU	–
1	Max pooling 1D	–	–	–	Size 2
1	Flatten	–	–	–	–
1	Dense	{10, 32, 64}	–	ReLU	–
1	Dropout	–	- -	–	Probability 0.1
1	Dense	{10, 32, 64}	–	ReLU	–
1	Dropout	–	–	–	Probability 0.1
1	Dense	1	–	Sigmoid	–
**Learning parameters**					
Learning rate	0.002				
Batch size	256				
Optimizer	Nadam				
Epochs	100				

Layers	Hyperparameter space	Activation			
**C: Bayesian-CNN (hg38 dataset)**					
No. of convolutional groups	$[0 \ldots 2]$				
No. of hidden convolutional layers, composing the group	$\{0, \ldots , 3\}$	ReLU			
No. of filters in the convolutional layer	$[0 \ldots 128]$				
2D kernel size in the convolutional layer	$[2 \ldots 8] \times [1,2]$				
Max pooling 2D	$[1 \ldots 8] \times [1,2]$				
Dropout	$[0 \ldots 0.5]$				
No. of dense groups	$[0 \ldots 2]$				
No. of hidden dense layers, composing the group	$\{0, \ldots , 3\}$	ReLU			
No. of units in dense layer	$[0 \ldots 64]$				
Dropout	$[0 \ldots 0.5]$				
Output	1	Sigmoid			
**Learning parameters**					
Learning rate	0.002				
l1 regularizer	0.0001				
l2 regularizer	0.0001				
Batch size	256				
Optimizer	Nadam				
Epochs	100				

In Tables B and C, for each otpimized hyperparameter, the search hyperparameter space is shown, where square brackets are used for continuous hyperparameter spaces, while curly brackets are used for discrete ones. “Max Pooling 1D” and “Max Pooling 2D” refer, respectively, to max-pooling 1D and 2D layers, “Average Pooling 1D” refers to average-pooling 1D layer, “Dropout” refer to dropout layers, and “Batch Norm” refers to batch normalization layer

### DeepEnhancer

To compare the *Bayesian-CNN* model to a state-of-the-art model, we implemented the 4conv2pool4norm DeepEnhancer network [33]. 4conv2pool4norm recognizes enhancers from background sequences by processing, one-hot-encoded genomic sequences through a 1D-CNN with four convolutional layers, where the input sequence has a window size W = 300, the first two convolutional layers have 128 1D-kernels (window size = $1 \times 8$) while the third and fourth layers have 64 1D-kernels (window size = $1 \times 3$); all layers are followed by a batch normalization layer, while a max-pooling layer is applied only after the second and the fourth layers. After the fourth layer, two dense layers with ReLU activation (256 and 128 neurons, respectively), and interleaved with a dropout layer (ratio 0.5), perform the processing that brings it to an output layer with two neurons and softmax activation, so that each neuron is regarded as a probability score predictor for one class (enhancer versus background sequences). Of note, to prove the effectiveness of the proposed architecture, in [33] authors present experiments by using new DeepEnhancer models obtained by: removing the batch normalization layer (4conv2pool model); removing the batch normalization and the max-pooling layer (4conv model); adding two more convolutional layers with 16 1D kernels (window size = $1 \times 2$) to both the 4conv2pool4norm and the 4conv2pool, therefore obtaining 6conv3pool6norm and 6conv3pool. All the comparison showed that 4conv2pool4norm is the best performing architecture.

In our experiments, the implemented 4conv2pool4norm model was used to process the same one-hot encoded sequence data (“Datasets” section) used to train and test our CNN models, and the 2-way output layer was substituted with an output layer composed by a single neuron with sigmoid activation, in line with our FFNN and CNN models. The implemented 4conv2pool4norm model was then trained and tested for each of the CRR activity prediction tasks detailed in “Experimental setup” section where training exploited the hyperparameters detailed in [33] for the 4conv2pool4norm: Adam optimizer [81], an initial learning rate set to $10^{-4}$, which decreases according to learning rate decay, a maximum of 30 epochs with an early stopping strategy to speed up the training.

## Datasets

In this Section, we detail the datasets for genome version hg19/GRCh37 (hg19-dataset, “Hg19-dataset” section) and for genome version hg38/GRCh38 (hg38-dataset, “Hg38-dataset” section).

### Hg19-dataset

The dataset for genome version hg19 contains regions belonging to GM12878, HelaS3, HepG2, K562 cell lines, where enhancers and promoters were labeled based on tags per million (TPM) data in the Cap Analysis of Gene Expression (CAGE) dataset downloaded from FANTOM5 [31]. In particular, the following labels were set, which refer to transcriptionally active or inactive enhancers and promoters:

- **AE** and **IE** indicate that the regions is, respectively, an *active* or *inactive enhancer*, where *active* means that the enhancer is transcribed with (TPM>0).
- **AP** and **IP** are assigned to *active* or *inactive promoters*, where an active (inactive) promoter is defined when TPM > 5 (TPM=0).
- **AX** and **IX** identify *active* or *inactive exons*, respectively. The class of the exon was defined based on exon transcription levels from RNA-seq data downloaded from ENCODE. In particular, if the peak-max of the exon is greater than 400 the label is AX, the exon is inactive (IX) if it is equal to zero.
- **UK** is the label for unknown/uncharacterized exons and DNase I open regions not contained in FANTOM5.

Under this setting, epigenomic data for training FFNN models were extracted from the ENCODE project [19], and CpG islands and phastCons scores were extracted by computing the mean value of the feature signal which falls within a 200 bps bin centered at each labelled region. Since the activity of CRRs is strongly influenced by chromosome accessibility (regulated through the interplay between histone modifications and binding of specific TFs) [82], histone and TFs ChIP-seq profiles, open chromatin (DNase-seq, FAIRE-seq) data and chromatin conformation (ChIA-PET) data were used as epigenomic features in this study (see ENCODE Data Standards^2^ for an overview about the mentioned high-throughput technologies). Moreover, phastCons and CpG island scores were considered because CRRs are known to be evolutionary conserved [83] and levels of DNA methylation are predictive of CRRs [84]. Such data is the same used by DECRES FFNN [34] and were provided by DECRES authors themselves.^3^

The CNN models (“Methods” section) were trained and tested by using genomic sequence data obtained for genome version hg19 from the UCSC repository dataset^4^ In particular, each genomic region is represented by a sequence of 200 nucleotides, which might be the usual A, C, G, and T nucleotides, or an unknown nucleotide (identified by N). The obtained 200 bps sequences were encoded using the one-hot encoding scheme.

To provide an overview of the unbalanced class distribution in each cell line, the top part of Table 1 shows the overall cardinality and the class distribution for each cell line from hg19. The available cell lines differ in sample cardinalities, with class AE being always the less represented (cardinality of AE is $5\%$ of the cardinality of IE, $16\%$ of AP, and $2\%$ of IP).

### Hg38-dataset

To validate our results on genome version hg38, we used a pipeline similar to that presented for hg19 (“Hg19-dataset” section) and downloaded only regions catalogued as enhancers and promoters from FANTOM5 for three of the cell lines used for hg19 (GM12878, HepG2, K562). We discarded the HelaS3 cell line, whose epigenomic features in ENCODE were mostly deprecated. In this case, we downloaded epigenomic data from ENCODE reflecting again the need to use data describing histone and TFs (ChIP-seq) profiles, open chromatin states (DNase-seq, ATAC-seq) and DNA methylation information (WGBS). Unfortunately, FAIRE-seq, ChIA-PET, CpG and phastCons data were not available from ENCODE for the hg38 genome version, however we added to our dataset further profiles describing open chromatin regions (ATAC-seq) and DNA methylation (WGBS). Interested readers can refer to ENCODE Data Standards ^[2]^ for details about the mentioned omics technologies. To label activity in the downloaded CRRs, similar to the hg19 dataset, we thresholded the TPM values. In line with the hg19 dataset [34], active (inactive) enhancers were defined as enhancers with TPM $>0$ (TPM $= 0$). Active (inactive) promoters were defined as promoters with TPM $> 5$ (TPM $\le 5$), which differs from the thresholding strategy used for the hg19 dataset, where regions with 0 < TPM $\le 5$ had undefined activity (“Hg19-dataset” section). In this way, we obtained active enhancers (AE), active promoters (AP), inactive enhancers (IE), and inactive promoters (IE) for genome version hg38. Under this setting, epigenomic features for genome version hg38 were downloaded from ENCODE,^5^ while the genomic sequence was downloaded from the UCSC repository.^6^

To provide an overview of the new class distribution for the available cell lines, we show the total sample cardinality and the cardinality of each class (AE, AP, IE, IP) for each cell line at the bottom of Table 1. Differently from hg19, the cell lines in hg38-dataset have equal sample cardinalities.

In particular hg19 dataset has, on average, the $79\%$ of enhancers and promoters with respect to hg38-dataset (comparison between rows *“Total E+P”* in Table 1). Further, Wilcoxon signed-rank test (*p* value $p < 0.01$) confirmed the statistically significant difference between the distributions of TPM values (tags per million, “Datasets” section) composed by samples present in both datasets, which is reflected in differences in the sample-activity labeling. In particular, when considering samples active in one or both datasets, $12\%$ of them have opposite activity labels.

To automate the genome assembly retrieval process, we developed a GitHub package available at https://github.com/LucaCappelletti94/ucsc_genomes_downloader, which is also available through the UCSC repository at https://genome-euro.ucsc.edu/util.html.

## Data Availability

The Python software library that implements the *Bayesian-FFNN*  and the *Bayesian-CNN*  models and the experiments performed in this paper are available in the GitHub repository at https://github.com/AnacletoLAB/crr_prediction.The pipeline that retrieves and combines the data relative to the hg38 human genome assembly are available at https://github.com/AnacletoLAB/epigenomic_dataset. The data relative to hg19 human genome assembly from [34] are partially available at https://github.com/yifeng-li/DECRES while the full data was made available to us upon request. The neural network configurations, as well as the performance and training history of all the evaluated models for all the holdouts are entirely available at: https://github.com/AnacletoLAB/crr_prediction/tree/main/results.

## Abbreviations

CRR: Cis-regulatory region
DNA: Deoxyribonucleic acid
GWAS: Genome-wide association studies
ML: Machine learning
FFNN: Feed forward neural network
CNN: Convolutional neural network
CAGE: Cap Analysis of Gene Expression
RNA: Ribonucleic acid
DECRES: DEep learning for identifying Cis-Regulatory ElementS
AE: Active enhancer
IE: Inactive enhancer
AP: Active promoter
IP: Inactive promoter
AX: Active exon
IX: Inactive exon
UK: UnKnown/uncharacterized
AUROC: Area Under the Receiver-Operating Curve
AUPRC: Area Under the Precision-Recall Curve
t-SNE: t-distributed Stochastic Neighbor Embedding

## Footnotes

1 For computing the t-SNE we used the tsne-Cuda implementation available at https://github.com/CannyLab/tsne-cuda.

2 ENCODE Data Standards at https://www.encodeproject.org/data-standards/.

3 ENCODE Data at ftp://hgdownload.cse.ucsc.edu/goldenPath/hg19/encodeDCC; ENCODE Fold-Change Data is described here https://sites.google.com/site/anshulkundaje.

4 UCSC repository dataset at https://genome.ucsc.edu/.

5 ENCODE Data at ftp://hgdownload.cse.ucsc.edu/goldenPath/hg38/encodeDCC.

6 UCSC repository dataset at https://genome.ucsc.edu/.
